# Growth Conditions Influence *Lactobacillus* Cell-Free Supernatant Impact on Viability, Biofilm Formation, and Co-Aggregation of the Oral Periodontopathogens *Fusobacterium nucleatum* and *Porphyromonas gingivalis*

**DOI:** 10.3390/biomedicines11030859

**Published:** 2023-03-11

**Authors:** Paola Zanetta, Diletta Francesca Squarzanti, Alessia di Coste, Angela Amoruso, Marco Pane, Barbara Azzimonti

**Affiliations:** 1 Laboratory of Applied Microbiology, Department of Health Sciences (DiSS), Center for Translational Research on Allergic and Autoimmune Diseases (CAAD), School of Medicine, Università del Piemonte Orientale (UPO), Corso Trieste 15/A, 28100 Novara, Italy; 2 Probiotical Research S.r.l., Via Mattei 3, 28100 Novara, Italy

**Keywords:** *Fusobacterium nucleatum*, *Porphyromonas gingivalis*, periodontopathogens, *Lactobacillus*, probiotics, probiotic cell-free supernatant, oral cancer prevention, oral squamous cell carcinoma

## Abstract

*Fusobacterium nucleatum* and *Porphyromonas gingivalis* human periodontopathogens play a leading part in oral squamous cell carcinoma through cell proliferation, invasion, and persistent inflammation promotion and maintenance. To explore how the activity of *Lactobacillus*-derived cell-free supernatants (CFSs) can be influenced by growth medium components, CFSs were produced both in the standard MRS and the novel animal-derivative-free “Terreno Industriale Lattobacilli” (TIL) media, and in vitro screened for the containment of *F. nucleatum* and *P. gingivalis* both single and co-cultured and also for the interference on their co-aggregation. The viability assay demonstrated that the *Limosilactobacillus reuteri* LRE11 and *Ligilactobacillus salivarius* LS03 MRS-produced CFSs were significantly more effective against single and co-cultured pathogens. All the other CFSs significantly improved their efficacy when produced in TIL. Both MRS- and TIL-produced CFSs significantly inhibited the single and co-cultured pathogen biofilm formation. Only *Levilactobacillus brevis* LBR01 CFS in MRS specifically reduced *F. nucleatum* and *P. gingivalis* co-aggregation, while viable LBR01, LS03, and LRE11 in MRS significantly co-aggregated with the pathogens, but only LS03 cultivated in TIL improved this effect. This work paves the way to better consider environmental growth conditions when screening for probiotic and postbiotic efficacy as crucial to pathogen aggregation, adhesion to the host’s niches, and exclusion.

## 1. Introduction

Oral microbiota eubiosis is crucial not only to avoid the onset of diseases such as caries, gingivitis, and periodontitis, but also to prevent the development and worsening of local and systemic pre-tumoral conditions [1].

Bacteria such as Gram-negative *F. nucleatum*, anaerobic oral commensal belonging to the phylum Fusobacteria have been historically known for their key role as periodontal pathogens in oral health biofilm structure and ecology [2]. The most accredited hypothesis also regarding its primary role in OSCC occurrence was first suggested by Katz and colleagues in 2011 who retrieved it in multispecies biofilm within OSCC lesions [3]. Its natural propensity to create connections between early and late bacterial colonizers resides in Fap2, a wall outer membrane adhesin, which mediates the biofilm formation [4]. Interestingly, it favors the adhesion of other periodontopathogens such as *P. gingivalis* by protecting it from low pH through glutamate and aspartate fermentation, thus producing alkaline ammonia components. Moreover, it increases its invasiveness in primary human gingival keratinocytes in vitro, inhibits the host’s innate immune response and induces carcinogenesis through cell epithelial–mesenchymal transition and apoptosis inhibition [5]. Finally, it promotes proinflammatory interleukins, tumor necrosis factor (TNF)-α, matrix metalloproteinase (MMP) production and DNA damage, inhibits apoptosis, and prevents p53 antitumoral activity [6,7,8]. 

In dysbiosis-promoting conditions, represented by an unbalanced and industrial diet, alcohol consumption, and smoking, this synergic co-dependent activity makes them identify as a pathogenic unit, rather than individual species, which thus further favors each of them in number and virulence [9,10,11]. Given the growing antibiotic resistance of the periodontopathogens that affect the patient’s global health, it is mandatory to reduce the administration of antibiotics. The development of novel strategies able to control this phenomenon must also take into account that their efficacy is mainly conditioned by the dynamic individual environmental context in which they are employed [12].

For these reasons, probiotics, other than helping eubiosis retrieval, are employed as powerful tools to control oral pathobiont growth and invasiveness, avoiding antibiotic misuse, and the development of further resistances. Thus, the cell-free supernatants (CFSs) of six *Lactobacillus* probiotic strains (*Levilactobacillus brevis* LBR01, *Ligilactobacillus salivarius* LS03, *Limosilactobacillus reuteri* LRE11, *Lacticaseibacillus rhamnosus* LR04, *Lacticaseibacillus casei* LC04, and *Limosilactobacillus fermentum* LF26) grown in two different media, the standard De Man, Rogosa, and Sharpe (MRS) and the novel animal-derivative-free, referred to generically as “Terreno Industriale Lattobacilli” (TIL), were produced. CFSs were then investigated to explore whether their capability to contain two emblematic commercial *F. nucleatum* and *P. gingivalis* periodontopathogenic strain viability and biofilm formation could be influenced by the medium components in which they were made, also validating their effect against the single and the co-cultured pathogen. The single pathogen and probiotic strain interactions and the probiotic CFS interference with the two-pathogen co-aggregation were also investigated, with auto- and co-aggregation assays in complex in vitro pathogen co-culture environments. The results obtained by this analysis demonstrate how environmental conditions are crucial to determine bacteria aggregation, interaction, the host’s niche adhesion and colonization, and the probiotic strain-mediated pathogen exclusion as it also happens in vivo.

## 2. Materials and Methods

### 2.1. Bacterial Cultures and Growth Curves

The periodontal pathogens *Fusobacterium nucleatum* (DSM 15643, Deutsche Sammlung von Mikroorganismen und Zellkulturen, DSMZ, Braunschweig, Germany) and *Porphyromonas gingivalis* (DSM 20709), respectively, isolated from cervico-facial lesion and human gingival sulcus, were cultivated at 37 °C in anaerobic 2.5 L rectangular jars with Oxoid^TM^ AnaeroGen^TM^ sachets (Thermo Fisher Diagnostic S.p.A., Rodano, Milan, Italy) overnight (ON) and using brain hearth infusion broth (BHI, Sigma-Aldrich, St. Louis, MO, USA, distributed by Merck Life Science S.r.l., Milan, Italy) supplemented with 0.5% N-acetyl-L-cysteine (Sigma-Aldrich), 5 µg/mL hemin (Sigma-Aldrich), and 0.5 µg/mL menadione (Sigma-Aldrich). Before each experiment, all the bacterial strains were freshly renewed.

The probiotic strains Levilactobacillus brevis LBR01 (DSM 23034), Ligilactobacillus salivarius LS03 (DSM 22776), Limosilactobacillus reuteri LRE11 (DSM 33827), Lacticaseibacillus rhamnosus LR04 (DSM 16605), Lacticaseibacillus casei LC04 (DSM 33400), Limosilactobacillus fermentum LF26 (DSM 33402) were aerobically grown in static conditions ON at 37 °C, using De Man, Rogosa, and Sharpe broth (MRS, Condalab, distributed by Cabru S.A.S., Biassono, Italy) and the animal derivative-free referred to generically as “Terreno Industriale Lattobacilli” (TIL) broth (Probiotical Research S.r.l., Novara, Italy; formula in g/L: proteose peptone N-3 10, dextrose 20, dipotassium phosphate 2, magnesium sulfate 0.1, manganese sulfate 0.05, vegetal extract—confidential, sodium acetate 5, Tween-80 1, yeast extract 5, ammonium citrate 2), containing peptones from plant sources, supplemented with fructose for LBR01 and glucose for the other probiotics. A growth curve was assessed for each probiotic strain in the standard MRS and in the novel TIL media by an optical density reading at 600 nm (OD_600_) using the NanoPhotometer NP80 (Implen, Munich, Germany). All probiotic strains were kindly provided by Probiotical Research S.r.l. 

### 2.2. Probiotic Cell-Free Supernatant Production

Probiotic cell-free supernatants (CFSs) were produced as previously described [13]. Fresh probiotic cultures were inoculated with an optical density at 600 nm (OD_600_) = 0.05 into MRS or TIL and grown ON. Bacterial growth was determined via OD_600_ measurement, then the supernatant was collected by centrifugating the bacterial cultures at 3000× *g* for 20 min at 4 °C (Heraeus Megafuge 16R, Thermo Fisher Scientific, Rodano, Milan, Italy). Then, the CFS were sterilized with 0.22 µm polyethersulfone (PES) filters (Clearline, distributed by Biosigma, Cona, Venice, Italy), aliquoted, and stored at −20 °C. MRS, TIL with fructose, and TIL with glucose were incubated as the probiotic cultures and used as controls in the following experiments (iMRS, iTILF, and iTILG, respectively). CFS pH was also determined (Sension + PH3, Hach Lange S.r.l., Milan, Italy).

### 2.3. Viability Assay

Pathogen viability after CFS treatment was assessed as previously described, with few modifications [13]. Pathogens were plated independently at OD_600_ = 0.05 in BHI (approximately 5 × 10^7^ CFU/mL) into a 96-well plate, CFS treatment was immediately added to the bacterial culture to obtain a final volume/volume ratio of 50%, and then the plate was incubated in anaerobic conditions at 37 °C. The viability assay was performed using the BacTiter-Glo^TM^ Microbial Cell Viability Assay (Promega Italia S.r.l., Milan, Italy), following the manufacturer’s instructions, at the time points of 24, 48, and 72 h. A Spark microplate reader (Tecan Trading AG, Switzerland) was used to detect luminescence. The pathogen co-culture was used to develop a complex viability assay. Before CFS treatment, pathogens were plated together at the same OD_600_ = 0.05, and the experiment was conducted as described above. In all of the experiments, BHI, iMRS, iTILF, and iTILG were used as controls. Each experiment was repeated three times independently and conducted with four replicates.

### 2.4. Biofilm Formation Assay

The biofilm formation of CFS-treated pathogens was determined through crystal violet (CV) staining, as previously described, with few modifications [13]. Pathogens were plated independently at OD_600_ = 0.05 into a 48-well plate and immediately treated with probiotic CFSs (50% *v*/*v*). OD_600_ was read before incubation (T_0_) and at 72 h, the time selected to obtain a mature biofilm. Then, 4% paraformaldehyde (Bio-Optica S.p.A., Milan, Italy) was used to fix the biofilm for 30 min at RT. The supernatant was removed, and the biofilm was stained with 1% CV solution (Sigma-Aldrich) for 15 min at RT. After removing the excess CV, the biofilm images were acquired on an EVOS FLoid^TM^ Cell Imaging Station (Thermo Fisher Scientific, Waltham, MA, USA). To quantify the biofilm amount, 33% acetic acid solution was used to dissolve the CV and the absorbance was read at 570 nm with a Spark microplate reader. The pathogen co-culture was used to develop a complex viability assay. Before CFS treatment, the pathogens were plated together at the same OD_600_ = 0.05 before CFS treatment; then, the assay was performed as described above. In all of the experiments, BHI, iMRS, iTILF, and iTILG were used as controls. Each experiment was repeated three times independently and conducted with four replicates.

### 2.5. Co-Aggregation Assay

Auto- and co-aggregation assays were used to study the bacterial interactions, as previously described, with few modifications [13]. Fresh ON pathogen and probiotic cultures were centrifuged at 3000× *g* for 15 min at RT (Heraeus Megafuge 16R). The pellet was resuspended at OD_600_ = 1 in a co-aggregation buffer (CAB; 150 mM NaCl, 1 mM Tris, 0.1 mM CaCl_2_, 0.1 mM MgCl_2_ 2H_2_O). Aliquots of 1 mL were prepared with equal amounts of pathogens, or pathogens and probiotics, to determine their co-aggregation, while the auto-aggregation was assessed using aliquots with only one bacterial suspension. Immediately (T_0_) and after 4 h (T_4_) incubation at RT, the OD_600_ of each aliquot was read (NanoPhotometer NP80; Implen). To inhibit the pathogen interactions, CFSs were also used (50% *v*/*v* with the pathogen mixture). As controls, the following conditions were used: pathogen and probiotic auto-aggregation values; D-glucose (D-glu), D-galactose (D-gal), and D-fructose (D-fru) solution in CAB (50 mM final) as the positive controls; Tween-20 0.05% in 0.2 M NaCl as the negative control (C−), to control for non-specific bacterial interactions [14]. The following equation was used to calculate the aggregation percentages:

$$\mathrm{auto}-\;\mathrm{or}\;\mathrm{co}-\mathrm{aggregation}\;\%\;=\;\frac{{\mathrm{OD}}_{600}{\;T}_{0}{-\mathrm{OD}}_{600}T_{4}}{{\mathrm{OD}}_{600}{\;T}_{0}}\;\cdot 100$$

with OD_600_ T_0_ = OD at T_0_ and OD_600_ T_4_ = OD at T_4_. Each experiment was conducted with three replicates and independently repeated three times.

### 2.6. Statistical Analysis

One-way and two-way ANOVA tests, with Tukey post-hoc correction, were performed using GraphPad Prism version 6.01 for Windows (GraphPad Software, San Diego, CA, USA, (accessed on 15 June 2018)). Results were represented as the mean of the replicates ± standard deviation (SD). Significant differences were considered for *p* < 0.05.

## 3. Results and Discussion

### 3.1. Probiotic Growth Curves

**Figure 1:**
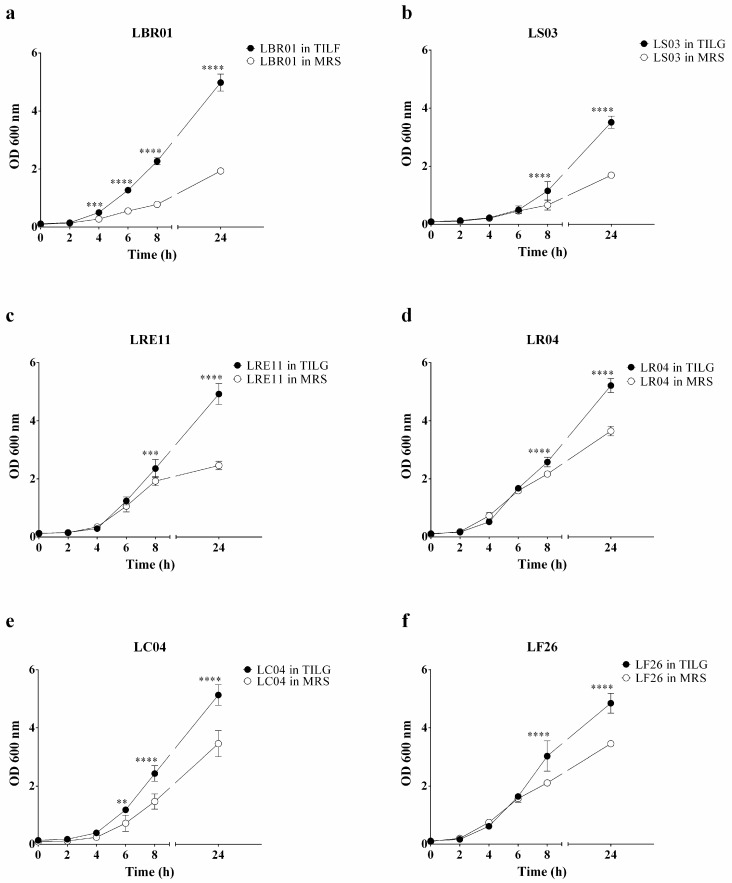
Probiotic growth curves. Probiotic growth curves in MRS and TIL media of (**a**) LBR01, (**b**) LS03, (**c**) LRE11, (**d**) LR04, (**e**) LC04, and (**f**) LF26. Data are expressed as the mean of three independent experiments ± SD. OD 600 nm = optical density at 600 nm. ** *p* < 0.01; *** *p* < 0.001; **** *p* < 0.0001.

Probiotic growth curves were conducted for each strain in the standard MRS and the novel animal-derivative-free TIL media. In Figure 1, the growth curves of all of the probiotics used are reported. All probiotics adapted well to the TIL medium, which could be observed in the graphs, where enhanced growth during the exponential phase is shown (Figure 1). Specifically, LBR01 had a significant growth increase in TIL when compared to MRS already after 4 h of incubation (*p* < 0.001; Figure 1a), with a further enhancement over time (*p* < 0.0001 at 6, 8, and 24 h; Figure 1a). LC04 showed a similar pattern, but with a mild variation at 6 h (*p* < 0.01; Figure 1e), which increased over time (*p* < 0.0001 at 8 and 24 h; Figure 1e), while LRE11 displayed growth dissimilarities only after 8 h of incubation (*p* < 0.001, and *p* < 0.0001 at 24 h; Figure 1c). LS03 had a significant growth increase in TIL after 8 h of incubation (*p* < 0.0001; Figure 1b) as well as LR04 (*p* < 0.0001; Figure 1d) and LF26 (*p* < 0.0001; Figure 1f), which was maintained until 24 h. In conclusion, the growth curves demonstrated that the tested probiotics could adapt and duplicate in a medium free from ingredients of animal origin. Moreover, their growth was even enhanced in TIL. However, this outcome can be probiotic strain specific; in fact, as shown by Squarzanti et al., other types of *Lactobacillus* did not show any significant growth difference when cultivated in MRS or TIL [15]. 

### 3.2. Viability Assay

Probiotic CFSs were first employed to determine their efficacy against the viability of each pathogen by using the highly sensible Bact-Titer Glo^TM^ Microbial Cell Viability Assay, which allows one to assess the viable pathogen cell number by quantifying the ATP present through the luminescent signal measurement, proportional to both the ATP amount and the viable bacterial cell number from as few as 10 bacterial cells. The CFS pH was on average 4.0.

**Figure 2:**
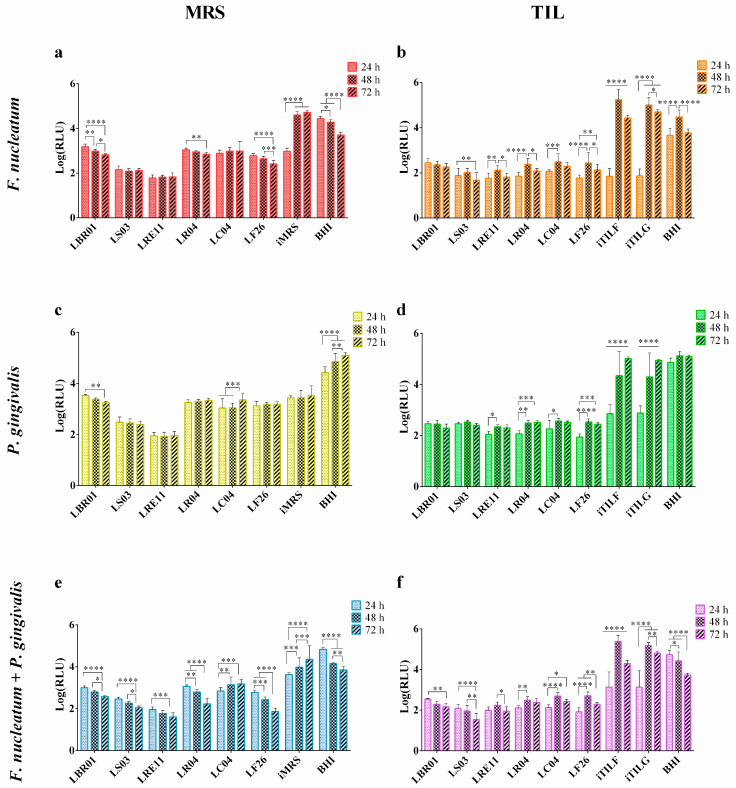
Viability assay. Pathogen viability was determined after 24, 48, and 72 h of probiotic CFS treatment in MRS: (**a**) *F. nucleatum*, (**c**) *P. gingivalis*, (**e**) *F. nucleatum* + *P. gingivalis*; and in TIL: (**b**) *F. nucleatum*, (**d**) *P. gingivalis*, (**f**) *F. nucleatum* + *P. gingivalis*. Data are represented as the Log(mean) of three independent experiments ± SD. * *p* < 0.05; ** *p* < 0.01; *** *p* < 0.001; **** *p* < 0.0001. Log(RLU) = Logarithm10 (relative luminescence unit); CFS = cell-free supernatant.

LRE11 CFS showed the highest inhibitory activity against *F. nucleatum* when cultivated in MRS, maintaining the same efficacy over time, and showing significant differences when compared to the other CFSs and the controls (*p* < 0.0001 vs. all conditions at all time points, except *p* < 0.001 vs. LS03 at 48 and 72 h; Figure 2a), followed by LS03 CFS (*p* < 0.0001 against all conditions at all endpoints; Figure 2a). LBR01, LR04, and LF26 CFSs improved their efficacy over time with the significant differences shown in Figure 2a. Among them, LF26 CFS was the one showing the best pathogen viability reduction at all time points (*p* < 0.0001, except *p* < 0.01 vs. LR04 at 24 h; Figure 2a). Despite *F. nucleatum* showing a significantly lower viability in iMRS at 24 h when compared to the BHI control (*p* < 0.0001; Figure 2a), probably due to the bacterium longer adaptation phase to the iMRS medium components, however, it significantly increased at 48 and 72 h (*p* < 0.0001; Figure 2a), fully recovering its growth performance. When probiotic CFSs were produced in TIL, no significant differences were observed at 24 h among the TIL controls and the CFS treatments, except for LBR01, where the pathogen viability was higher (*p* < 0.0001 vs. all conditions, but *p* < 0.05 vs. LC04; Figure 2b); conversely, a significant difference was obtained comparing all of these conditions to BHI (*p* < 0.0001; Figure 2b). At 48 and 72 h, all probiotics showed the same significant difference against all of the controls (*p* < 0.0001; Figure 2b). In this case, all probiotic CFSs displayed the same activity without any significant difference, apart from LRE11 CFS, which showed a significant difference against that of LC04 of *p* < 0.05 at 48 h and *p* < 0.001 at 72 h, and vs. LBR01 CFS at 72 h (*p* < 0.001; Figure 2b). Another exception was LS03 CFS, which showed at 48 h, a *p* < 0.05 vs. LBR01 and LR04 CFSs, *p* < 0.01 vs. LF26 CFS, *p* < 0.001 vs. LC04 CFS, while at 72 h, these differences increased (*p* < 0.01 vs. LR04 CFS, *p* < 0.001 vs. LF26 and LRE11 CFSs, *p* < 0.0001 vs. LBR01 and LC04 CFS; Figure 2b). Furthermore, in this case, *F. nucleatum* showed significantly lower viability in the TIL controls at 24 h when compared to BHI (*p* < 0.0001; Figure 2a), with a significant increase at 48 and 72 h (Figure 2a, *p* < 0.0001). No significant differences were observed between iTILF and iTILG at all time points. When comparing the activity of the same probiotic CFS produced in the two media, it was noticed that the TIL-produced CFSs showed better efficacy compared to the MRS ones. In fact, LBR01, LR04, and LC04 CFSs significantly improved their efficacy when produced in TIL, showing a significant difference to the MRS-produced ones at all time points (*p* < 0.0001). LS03 and LF26 CFSs, instead, were more effective when produced in TIL only at 24 and 72 h, showing *p* < 0.001 and *p* < 0.0001 vs. LS03 CFS in MRS, and *p* < 0.0001 and *p* < 0.01 vs. LF26 CFS in MRS, respectively.

The efficacy pattern observed in *F. nucleatum* treated with CFSs produced in MRS is like the one observed in *P. gingivalis* (Figure 2c). LRE11 CFS still showed the best activity (*p* < 0.0001 vs. all conditions at all endpoints; Figure 2c), followed by that of LS03 (*p* < 0.0001 vs. all conditions at all time points; Figure 2c). It is unlikely that due to the sensibility of *P. gingivalis* to pH values below the neutrality, this pathogen did not adapt to the iMRS medium, in which it significantly showed lower viability than in BHI at all the times tested (*p* < 0.0001; Figure 2c) [16]. However, other CFSs also induced a significant reduction in *P. gingivalis* viability when compared to the iMRS control. Specifically, at 24 h, the LC04 and LF26 CFSs displayed respectively a *p* < 0.0001 and *p* < 0.01 vs. iMRS, maintained for LC04 CFS only at 48 h, while LF26 CFS was *p* < 0.05 (Figure 2c). At 72 h, LBR01 CFS reported a *p* < 0.05, while that of LF26 was *p* < 0.001 vs. iMRS (Figure 2c). Instead, despite the pH 6 of iTILF and iTILG being the same as that of iMRS, after an adaptation phase, *P. gingivalis* was able to grow in the iTILF and iTILG controls, even though their viability was still significantly lower than that in BHI until 48 h (*p* < 0.0001; Figure 2d). No differences were observed between the two iTILs at all time points. Probiotic CFSs showed a similar effect over time, with the LBR01 and LS03 CFSs being stable while those of LRE11, LR04, LC04, and LF26 showed a reduction in their activity, as shown in Figure 2d. At 24 h, only the LBR01 and LS03 CFSs showed significant differences against those of LRE11 (*p* < 0.05; Figure 2d), LR04 (*p* < 0.05; Figure 2d), and LF26 (*p* < 0.001; Figure 2d). At 48 and 72 h, instead, no differences among treatments were observed. TIL-produced CFSs also performed better against *P. gingivalis*. In fact, LBR01, LR04, LC04, and LF26 CFSs showed a significantly stronger reduction of the pathogen viability at all time points when compared to the MRS-produced ones (*p* < 0.0001). Conversely, the only difference for LS03 CFS was at 48 h, where, in this case, a better reduction was obtained with the CFS produced in MRS (*p* < 0.0001), while the same was found for LRE11 CFS with a *p* < 0.0001 at 48 and 72 h. 

When *F. nucleatum* and *P. gingivalis* were co-cultured, more heterogeneous results were observed when MRS-produced CFSs were used (Figure 2e). Furthermore, in this case, LRE11 CFS was the most effective, increasing its activity over time (*p* < 0.0001 vs. all conditions at all endpoints, except *p* < 0.001 vs. LS03 CFS at 48 h and no significant vs. LF26 CFS at 72 h; Figure 2e). The other probiotic CFSs also displayed an increased activity over time, except for that of LC04, which decreased, as shown in Figure 2e. When the TIL-produced CFSs were used, a similar pattern to the single bacteria was observed (Figure 2f). An adaptation phase to iTILs was still present but with a minor difference from BHI when the single pathogens were cultured, likely due to the protective activity of *F. nucleatum* toward *P. gingivalis*, which produces alkaline ammonia components, helps acquire essential nutrients, and produces virulence factors, as also observed by [17]. Nevertheless, all treatments showed a significant difference against the controls at all time points (*p* < 0.0001; Figure 2f). Between iTILF and iTILG, no significant difference was observed, except at 72 h (*p* < 0.001; Figure 2f). Both iTILs showed significant differences with BHI at all time points (*p* < 0.0001; Figure 2f). When LBR01 was produced in TIL, it significantly reduced the two-pathogen viability better than when produced in MRS at all time points (*p* < 0.0001). The same was seen for LS03 CFS, with *p* < 0.0001 at 24 and 72 h, *p* < 0.001 at 48 h, and LC04 CFS (*p* < 0.0001 at all endpoints). LRE11 CFS, instead, showed the same activity at 24 h when produced in both media, but it was more effective at 48 and 72 h when produced in MRS (*p* < 0.0001). LR04 CFS enhanced its effect when it was TIL-produced only at 24 and 48 h (*p* < 0.0001 and *p* < 0.00, respectively), while LF26 CFS was better in TIL only at 24 h (*p* < 0.0001), since it had the highest efficacy when produced in MRS at 48 and 72 h (*p* < 0.01 and *p* < 0.0001, respectively). 

To summarize, LRE11 CFS was the best in reducing both the single pathogen and their co-culture viability when cultivated in MRS, followed by that of LS03 only against the single *F. nucleatum* and *P. gingivalis*. This could likely be due to the advantage offered by reuterin, a broad-spectrum antimicrobial molecule produced by Lactobacillus reuteri strains during their anaerobic metabolism [18]. When produced in TIL, instead, LRE11 CFS did not show any improvement in its efficacy, still being the most effective when it was MRS-produced, while an improvement was observed for the LBR01, LR04, LC04, and LF26 CFSs. In the literature, other groups have also reported probiotic effects in the containment of oral pathogens, but only when produced in the standard MRS medium. In particular, many studies have highlighted the positive effect of *L. reuteri* strains such as the one from Jansen et al., which revealed how two strains of *L. reuteri*, ATCC PTA 5289 and DMS 17938, inhibited both *F. nucleatum* and *P. gingivalis* growth, but the PTA 5289 strain only when glycerol was added to the experiment [19]. Moreover, by combining the two *L. reuteri* strains, the glycerol effect was no longer relevant, and the pathogen inhibition was stronger [19]. In addition, *L. reuteri* ATCC PTA 5289 and DSM 17938 were used as viable, heat-killed, and CFS treatments against *F. nucleatum*, being able to significantly reduce its colony-forming unit (CFU) count [20]. The ability of live *L. reuteri* to benefit oral health is not new since Kang MS and collaborators in 2011 demonstrated that the human-derived KCTC 3594, KCTC 3678, and the rat-derived KCTC 3679 strains inhibited *Aggregatibacter actinomycetemcomitans*, *F. nucleatum*, *P. gingivalis*, and *Tannerella forsythia* growth, and *Streptococcus mutans* biofilm formation [21]. In their opinion, this activity was due to organic acids, hydrogen peroxide, and to the bacteriocin-like molecule reuterin, which also inhibits methyl mercaptan production, responsible for halitosis, by *F. nucleatum* and *P. gingivalis* [21,22]. Interestingly, about ten years later, Yang and colleagues demonstrated that the AN417 *L. reuteri* strain inhibited the viability and biofilm formation of *F. nucleatum* and *P. gingivalis* through carbohydrates and/or fatty acid metabolites. In fact, after the CFS digestion with α-amylase and lipase, this property was strongly reduced, especially toward *P. gingivalis*. Furthermore, the minimal inhibitory volume of the *L. reuteri* AN417 CFS able to reduce the pathogen growth rate and viability was 10% (*v/v*) against *P. gingivalis* and 20% (*v/v*) against *F. nucleatum* [23]. Mulla and colleagues also observed that *P. gingivalis* directly isolated from the patients’ dental plaque was sensitive to the treatment with the *L. reuteri* probiotic [24]. Other authors have demonstrated that the CFS of fermented sausage-derived *L. reuteri* strains also had antibacterial properties due to lactic acid and low pH, emphasizing once more the capability of each strain to exhibit specific metabolites [25]. 

Van Holm and colleagues also found that CFS from *L. rhamnosus* GG strongly killed *P. gingivalis*, while against *F. nucleatum*, *L. reuteri* F471 was more effective [26]. *L. rhamnosus* CT-53 inhibited *F. nucleatum* and *P. gingivalis* when used both as a viable strain in the agar overlay method, and heat-killed strain in the pathogen CFU counting after treatment [27]. It was also observed that milk fermented by *L. rhamnosus* I-1/13 as well as the probiotic viable culture, inhibited *P. gingivalis* growth in an agar well diffusion assay [28]. A similar method was also used by Gönczi et al., who found that *L. rhamnosus* and *L. casei* were effective strains for *F. nucleatum* and *P. gingivalis* containment [29]. The *L. fermentum* OK strain, when co-cultured with *F. nucleatum* and *P. gingivalis*, significantly inhibited their growth, although its proliferation was also retarded [30]. *L. fermentum* SG-A95 and *L. salivarius* SG-M6 were used both as viable strains and CFSs against *P. gingivalis*, being able to significantly reduce its growth in agar disc diffusion and broth dilution methods. Specifically, *L. fermentum* SG-A95 showed a stronger effect when used as a viable strain, while *L. salivarius* SG-M6 was when its CFS was used [31]. This evidence, together with our results, highlight not only how the probiotic effect is strain specific, but also that by changing the growth conditions, the efficacy of a specific strain can be different due to distinct by-products produced from the nutrients available [15]. In addition, Keller and colleagues in 2011 conducted a spectrophotometric analysis on a selection of *Lactobacillus* probiotics, indicating that *S. mutans* growth inhibition was strain specific and the pH and cell concentration was dependent, indicating once more that the response to therapy with lactobacilli-derived probiotics might vary among individuals and depend on the strain used [32]. 

### 3.3. Biofilm Formation Assay

**Figure 3:**
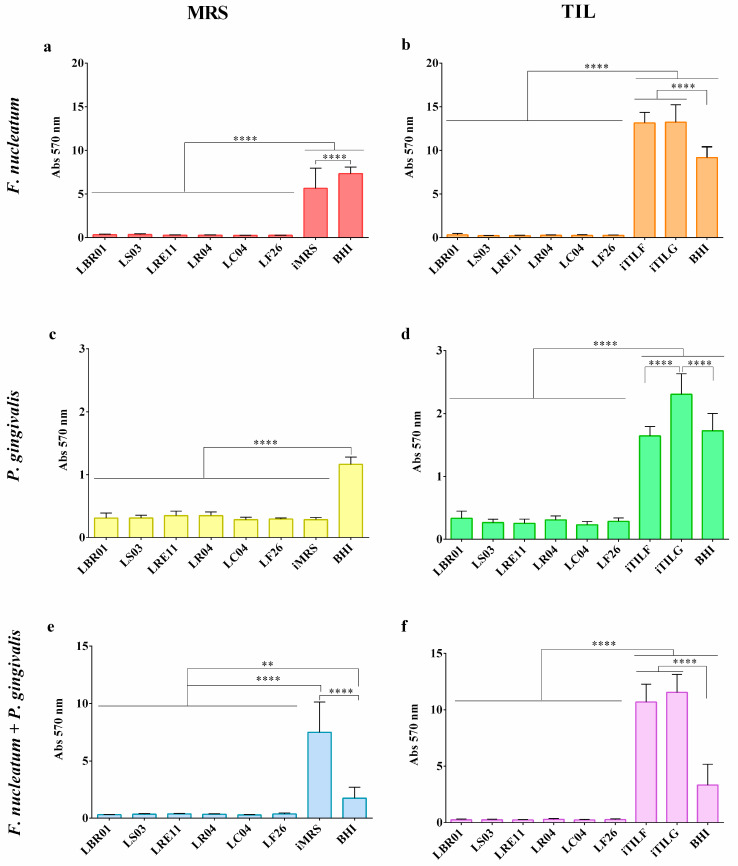
Crystal violet (CV) biofilm quantification. Pathogen biofilm quantification was determined after 72 h of probiotic CFS treatment in MRS: (**a**) *F. nucleatum*, (**c**) *P. gingivalis*, (**e**) *F. nucleatum* + *P. gingivalis*; and in TIL: (**b**) *F. nucleatum*, (**d**) *P. gingivalis*, (**f**) *F. nucleatum* + *P. gingivalis*. Data are represented as the mean of three independent experiments ± SD. ** *p* < 0.01; **** *p* < 0.0001. Abs 570 nm = absorbance at 570 nm; CFS = cell-free supernatant.

**Figure 4:**
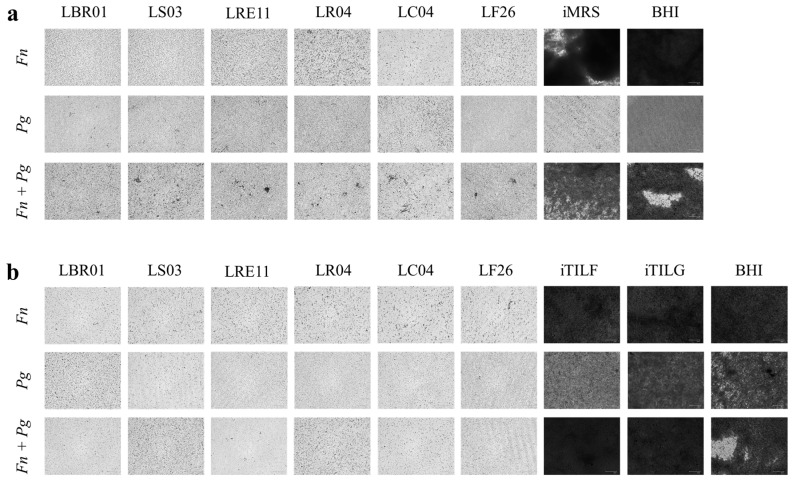
Representative crystal violet (CV) biofilm quantification. Representative CV-stained biofilm images. (**a**) MRS-produced CFS treatment; (**b**) TIL-produced CFS treatment. Images were obtained on a FLoid^TM^ Cell Imaging Station. Magnification 460×. *Fn*, *F. nucleatum*; *Pg*, *P. gingivalis*; *Fn* + *Pg*, *F. nucleatum* + *P. gingivalis*.

The ability of probiotic CFSs to reduce the biofilm formation was investigated. The OD_600_ measurement was used to assess pathogen growth at 72 h before performing the biofilm formation assay. The results of the CV-stained biofilm are reported in Figure 3. The *F. nucleatum* biofilm formation was significantly reduced when treated with probiotic CFSs both produced in MRS and TIL when compared to the controls (*p* < 0.0001; Figure 3a,b). iTILG and iTILF determined a higher biofilm formation compared to the BHI control (Figure 3b). LS03 CFS was more effective when produced in TIL, showing a significant difference when compared to LS03 CFS in MRS (*p* < 0.001). Against *P. gingivalis*, probiotic CFSs were also effective in reducing the biofilm amount with respect to the amount formed in the BHI control (*p* < 0.0001; Figure 3c,d). However, *P. gingivalis* did not form biofilm in iMRS, showing no difference to the CFS treatments (Figure 3c), while it did in iTILF and iTILG, showing significant differences not only with the CFSs, but also with the BHI control (*p* < 0.0001 TILG vs. BHI; Figure 3d). In this case, only the LRE11 CFS showed an increased efficacy when produced in TIL compared to MRS (*p* < 0.01). When *F. nucleatum* and *P. gingivalis* were co-cultured, all treatments showed significant differences to the controls (*p* < 0.01 vs. BHI, *p* < 0.0001 vs. TILF and TILG; Figure 3e,f); moreover, iMRS, iTILF, and iTILG promoted a higher biofilm formation when compared to the BHI control (*p* < 0.0001; Figure 3e,f). Against the pathogen co-culture, the TIL-produced LS03, LRE11, and LF26 CFSs showed an improved effect against biofilm formation compared to themselves produced in MRS (*p* < 0.001, *p* < 0.0001, *p* < 0.001, respectively). Figure 4 shows the representative CV-stained biofilm images. As was noticeable, the BHI results, among the different repetitions for each condition, were significantly different. In fact, while in BHI medium, the biofilm that formed was easier to come off, and was more consistent in the probiotic media used as the controls. For this reason, the BHI control was therefore repeated for each experiment. In our opinion, this evidence indicates that, in general, not only is there variability within the same species according to the strain type, but also that the same bacteria in the same growing conditions do not always grow and behave in an identical specific manner, as occurs in real life.

Van Holm et al. also observed that the presence of *L. rhamnosus* GG decreased the amount of *F. nucleatum* and *P. gingivalis* in their own biofilm [26]. *L. reuteri* AN417 CFS reduced the biofilm integrity of *F. nucleatum* and *P. gingivalis*, and also decreased the *P. gingivalis* expression of genes involved in biofilm formation [23]. *L. reuteri* DSM 17938 and *L. rhamnosus* HN001 Howaru^TM^ significantly decreased the biofilm formation of *P. gingivalis* together with *Streptococcus oralis* and *S. gordonii* also reducing its relative abundance [33]. In another in vitro analysis, pH-neutralized, catalase- or trypsin-treated CFSs from *L. reuteri* ATCC 23272 and *L. salivarius* ATCC 11741 were assessed to counteract *S. mutans* biofilm-, quorum sensing-, and stress survival-related gene expression, to identify the specific role of organic acids, peroxides, and bacteriocins. It was found that the antibacterial and antibiofilm activity of the CFSs was pH dependent. Moreover, *L. salivarius* showed the highest peroxide-dependent antibiofilm activity leading to a reduced production of exopolysaccharide matrix by the pathogen. In addition, the quantitative real-time polymerase chain reaction showed a decreased acid tolerance and quorum sensing-related gene expression [34].

### 3.4. Co-Aggregation Assay

**Figure 5:**
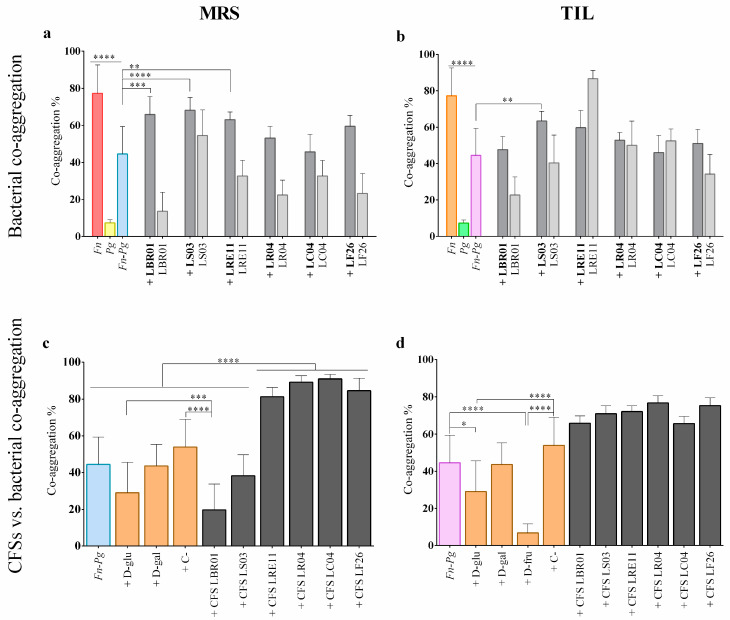
Auto- and co-aggregation assay executed with pathogens and probiotics grown in MRS (**a**) and TIL (**b**). The probiotic strain code alone refers to auto-aggregation; if a “+” before the probiotic strain is present, it means that the probiotic was added to the three-pathogen mix. CFS effects on the three-pathogen co-aggregation in MRS (**c**) and TIL (**d**). All data are represented as the mean of three independent experiments ± SD. * *p* < 0.05; ** *p* < 0.01; *** *p* < 0.001; **** *p* < 0.0001. *Fn* = *F. nucleatum*; *Pg* = *P. gingivalis*; D-glu = D-glucose; D-gal = D-galactose; D-fru = D-fructose; C- = negative control; CFS = cell-free supernatant.

Auto- and co-aggregation assays were performed to collect information on the single pathogen and probiotic strain interactions and to investigate whether probiotic CFSs produced in the two different media could differentially interfere with the two-pathogen co-aggregation. The auto-aggregation of each single bacterial strain, and the co-aggregation of the two pathogens with a probiotic strain, are shown in Figure 5a (probiotics grown in MRS) and Figure 5b (probiotics grown in TIL). *F. nucleatum* showed a strong auto-aggregation ability, while this was not present in *P. gingivalis*. Both the single pathogens exhibited a significant difference when compared to their co-aggregation (*p* < 0.0001; Figure 5a,b). The probiotic strains also showed a high auto-aggregation ability when cultivated in both media, but only in the conditions +LBR01, +LS03, and +LRE11 was the co-aggregation significantly increased when compared to the one of the two pathogens alone when grown in MRS (*p* < 0.001, *p* < 0.0001, *p* < 0.01, respectively). Only LS03 grown in TIL retained its ability to co-aggregate with the two pathogens (*p* < 0.01). Comparing the activity of the same probiotic cultivated in the two media, +LBR01 was the only condition showing a significant difference (*p* < 0.0001). The CFS effects on pathogen co-aggregation are shown in Figure 5c (MRS) and Figure 5d (TIL). Among the controls, D-glucose and D-fructose were the only ones showing a significant reduction in the pathogen co-aggregation (*p* < 0.05 and *p* < 0.0001, respectively; Figure 5d) that was also specific (*p* < 0.0001 for D-glucose and D-fructose vs. C-; Figure 5d). Among the probiotic CFSs, only those of LBR01 and LS03 in MRS significantly reduced the pathogen co-aggregation (*p* < 0.0001; Figure 5c), with only LBR01 CFS showing a specific activity (*p* < 0.0001 vs. C-; Figure 5c). All the other CFSs produced in MRS, instead, significantly increased the two-pathogen co-aggregation (*p* < 0.0001; Figure 5c). All TIL-produced CFSs significantly increased the pathogen co-aggregation (*p* < 0.0001, except *p* < 0.001 for LBR01 and LC04 CFSs; Figure 5d). However, for the TIL-produced LRE11, LR04, LC04, and LF26 CFSs, that increase was significantly lower than the one observed with CFS produced in MRS (*p* < 0.0001 for LC04, *p* < 0.001 for LR04, *p* < 0.05 for LRE11 and LF26). 

To our knowledge, this is the first paper reporting on viable probiotics and their CFS possible interactions with *F. nucleatum* and *P. gingivalis* co-aggregation. In fact, in the literature, it was only found that the *L. fermentum* OK strain co-aggregated efficiently with the single *P. gingivalis* pathogen [30].

Interestingly, in this assay, only LBR01 and LS03 CFSs in MRS were able to significantly inhibit the two-pathogen co-aggregation, with LBR01 CFS showing a specific inhibition since the co-aggregation percentage was lower than the negative control. A similar result was also obtained in our previous work against the oral pathogens *Aggregatibacter actinomycetemcomitans*, *Streptococcus mitis*, and *S. mutans* [13]. However, when these CFSs were TIL-produced, no one was able to significantly reduce the two-pathogen co-aggregation. This could be due to the different nutrients present in the two media, and thus in the metabolites that probiotics can produce according to the growth conditions. On the other hand, the LRE11, LR04, LC04, and LF26 CFSs significantly increased *F. nucleatum* and *P. gingivalis* co-aggregation, when both were MRA- and TIL-produced, even though that increase was lower for the CFSs in TIL. However, these probiotic CFSs were effective in reducing the viability and biofilm formation of the two pathogens. This difference may be due to the experimental designs, as already pointed out in our previous paper [13]. In fact, in the viability and biofilm formation assays, pathogens were plated in their elective medium and incubated in the optimal growth conditions, allowing for their adaptation to the CFSs added to the medium. Instead, in the co-aggregation assay, only molecule interactions were facilitated, since the bacteria were all resuspended into the CAB buffer. Moreover, the CFSs still contained some non-metabolized molecules of the *Lactobacillus* media, which may interfere with each other. This evidence once again highlights the importance of deeply investigating the CFS composition to identify the molecules in charge of a specific effect and isolate them from possible inhibitors, other than to characterize the specific metabolites produced in the two different media.

Viable probiotics can interact with pathogen cells by competing for cellular receptor binding and avoiding the cell adhesion of pathogens to human cells [35]. Malfa and collaborators investigated probiotic co-aggregation with urogenital pathogens, demonstrating that single and mixed *Lactobacillus* strains could prevent urogenital infections without impairing tissue integrity [36]. Zakaria et al. suggested that quorum sensing signaling molecules could explain the co-aggregation between *Lactobacillus* probiotics and pathogens, thus contributing to creating barriers that are able to prevent pathogen adhesion to epithelia and tissue invasion, and increasing the host defenses against infections in several districts [37]. Our results demonstrate that LBR01, LS03, and LRE11 viable cells significantly co-aggregated with *F. nucleatum* and *P. gingivalis* when cultivated in MRS, but only LS03 retained this activity when grown in TIL. We assumed that different growth media not only determine the CFS composition, but also that a different expression or function of the probiotic outer membrane molecules may be triggered. 

## 4. Conclusions

In conclusion, this work shows how different methodologies for probiotic CFS screening can be employed to investigate their potential for pathogen containment, allowing for the selection of the most promising ones. Furthermore, this research demonstrates how probiotic growth conditions can impact their CFS potential against oral pathogens, allowing for the identification of the best growth conditions to obtain the strongest and most useful effect. These experiments revealed that LRE11 and LS03 are the most promising strains able to counteract the growth and biofilm formation of *F. nucleatum* and *P. gingivalis*, paving the way for further and more specific studies on their CFS composition and the interaction with human oral cells infected by these two pathogens. Thus, the oral microbiota eubiosis imbalance, which also promotes oral carcinogenesis, can be broken by avoiding the use of antibiotics and increasing the antibiotic resistance, thus helping patients at risk in the prevention of cancer onset and progression. However, more experiments are needed including CFS mass spectrometry characterization and effect evaluation on the biofilm associate gene expression patterns before their employment in vivo in odontology in specific dysbiotic patient categories.

In this research work, we highlighted how culture conditions can influence bacterial potential and behavior, making it important to calibrate, at best, the environmental settings to obtain the maximum effect from probiotic metabolites. Additionally, after postbiotic pilot and screening studies such as this one, it is fundamental to focus the research on their postbiotic composition and their interaction with human oral infected cells. In this way, a more useful and delineated deployment on individuals will be determined, preserving oral eubiosis and avoiding antibiotic misuse and side effects. In addition, patients at risk of cancer development may find a valid ally to prevent, or at least delay, the onset and progression of this pathology.

## Data Availability

All relevant data are within the manuscript.
